# Evaluating multi-cancer early detection tests: an argument for the outcome of recurrence-updated stage

**DOI:** 10.1038/s41416-023-02434-4

**Published:** 2023-09-19

**Authors:** Matthew E. J. Callister, Emma J. Crosbie, Philip A. J. Crosbie, Hilary A. Robbins

**Affiliations:** 1 Consultant Respiratory Physician, Leeds Teaching Hospitals, Leeds, UK; 2 Honorary Professor of Respiratory Medicine, Leeds Institute of Health Sciences, University of Leeds, Leeds, UK; 3 National Institute for Health and Care Research Advanced Fellow, Professor and Honorary Consultant in Gynaecological Oncology, Division of Cancer Sciences, University of Manchester, Manchester, UK; 4 Professor of Respiratory Medicine, Division of Infection, Immunity and Respiratory Medicine, University of Manchester, Manchester, UK; 5 Scientist, Genomic Epidemiology Branch, International Agency for Research on Cancer, Lyon, France

**Keywords:** Randomized controlled trials, Cancer screening

## Abstract

The advent of multi-cancer early detection (MCED) tests has the potential to revolutionise the diagnosis of cancer, improving patient outcomes through early diagnosis and increased use of curative therapies. The ongoing NHS-Galleri trial is evaluating an MCED test developed by GRAIL, and is using as its primary endpoint the absolute incidence of late-stage cancer. Proponents of this outcome argue that if the test reduces the number of patients with advanced, incurable cancer, it can be reasonably assumed to be benefitting patients by reducing cancer mortality. Here, we argue that this assumption may not always hold due to the phenomenon of micro-metastatic disease, and propose an adjustment to the trial outcome so that it may better reflect the expected effect of the test on cancer mortality.

The potential widespread roll-out of MCED tests in nationwide screening programmes will be expensive and could carry harms that differ from traditional single-cancer screening. It is therefore imperative that any roll-out is supported by robust evidence for the test’s clinical efficacy and cost-effectiveness. The accepted gold-standard evidence for a cancer screening test is a randomised controlled trial demonstrating a reduction in cancer-specific mortality, as this avoids biases such as lead-time or overdiagnosis. However, trials powered for mortality outcomes need to be large, expensive, and can take a decade or more to report by which time the technology may be out of date. The recent advances in systemic anti-cancer treatment might exacerbate this problem, because if treatments are more effective at prolonging survival in patients with metastatic disease, then demonstrating a mortality benefit will require even longer follow-up. This has led to increased interest in surrogate endpoints which ideally would yield robust evidence of benefit over a much shorter follow-up period. This would have the dual advantage of allowing more rapid implementation to save lives, whilst also driving innovation by ensuring earlier return on investment for those developing new technologies.

The NHS-Galleri trial is a randomized controlled trial being conducted in partnership with the English National Health Service. The primary endpoint is the absolute incidence of late stage cancer, with the study powered at 90% to detect a reduction in stage III and IV cancers for all sites combined after three rounds of screening compared to usual care [1]. This endpoint was selected because it is not influenced by overdiagnosis, as it is unaffected by diagnosis of indolent disease. The study has recruited at a remarkable rate, exceeding its target of 140,000 participants in only 10 months [2]. The speed of recruitment, which has taken place in over 150 community locations, highlights the advantages of undertaking trials of this size in the NHS, with its centralised population databases and well curated cancer registry data. The NHS in England has committed to purchase 1 million tests for use in the English population if “initial results are promising” although no specifics have been provided for the criteria that will trigger this roll-out [2]. It will be important to collect detailed data on this initial implementation, which could allow assessment of MCED performance at scale in a real world setting.

The GRAIL Galleri test uses methylation patterns of cell-free DNA, released into the blood by apoptosis or necrosis, to detect a cancer signal. It has been shown that patients with cancer and a positive Grail MCED test, compared to those with a negative test, have worse overall survival, even when analysis is limited to early-stage cancers (i.e. stage I and II) [3]. There are two possible explanations for this phenomenon, which may co-exist. The first is that the MCED negative group includes some over-diagnosed cancers, i.e. cancers that would not have shortened the patient’s life even if left undetected and untreated. As the aim of MCED tests is to reduce cancer deaths, avoidance of detection of harmless cancers would be a strength of the test. An alternative explanation is that, even among potentially harmful cancers, MCEDs tend to identify cancers with increased risk of late recurrence. Such late recurrence is thought to be mediated by micro-metastatic disease—i.e. the cancer has spread to distant sites even at the time of initial diagnosis, but the metastatic deposits are too small to be detected by conventional staging investigations such as CT or PET-CT. The patient is erroneously diagnosed with an early stage cancer and undergoes curative-intent treatment (usually surgery) only for the disease to later recur and eventually shorten their life. If an MCED test preferentially detected such cancers, this would limit its ability to save lives through early detection.

The issue of micro-metastatic disease has implications for assessing the performance of a test in a randomised controlled trial if surrogate (non-mortality) outcomes are used. Consider a hypothetical example in which an MCED detects many asymptomatic cancers in participants in the intervention arm. Subsequent investigations find that the majority of these participants do not have detectable metastatic disease, are thus recorded in the cancer registry as early-stage cases and undergo curative-intent treatment. The test appears to have fulfilled its intended role by producing a reduction in the incidence of late-stage disease. Unfortunately though, the positive MCED test heralds micro-metastatic disease in every case, with subsequent incurable distant metastatic recurrence occurring after the initial staging assessment and treatment. Despite earlier detection, the course of the disease is unaltered, and patients die at the same time as they would have without the test. Although the test provides no overall clinical benefit, a study using the incidence of late-stage disease as its primary endpoint would still report a positive result.

Is there evidence of this phenomenon in other screening settings? The UK Collaborative Trial on Ovarian Cancer Screening (UKCTOCS) compared two screening strategies with no screening in over 200,000 women. After a median follow-up of 16 years, there was a 25% (95% CI −42% to −2%) reduction in the absolute incidence of stage IV disease in the multi-modal screening arm compared with no screening, but no significant reduction in ovarian and tubal cancer deaths [4]. The authors concluded that the cancers which were shifted to an earlier stage in the intervention arm probably had an intrinsic poor prognosis. One possible explanation is that metastatic cells are shed into the peritoneum at a very early stage in a cancer’s development - the so-called “precursor escape” phenomenon [5]. It may be that the additional early-stage cancers detected by screening in UKCTOCS had already progressed, but in a manner that was undetectable using standard staging approaches.

To address this potential problem and ensure that the NHS-Galleri trial can render the most clinically relevant answer possible, we suggest using an outcome which we call “recurrence-updated stage.” With this outcome, the trial would continue to assess the absolute incidence of late-stage disease as the primary outcome, but update the stage based on instances of cancer recurrence. Thus, in both study arms, the primary outcome would include participants who were initially diagnosed at stage III or IV, as well as any participants who were initially diagnosed at stage I or II but whose cancer later recurred in an anatomical location that would define stage III or IV depending on the appropriate TNM staging system (i.e. locally advanced or distant metastatic disease). Evidently, this would require standardized active follow-up of all participants diagnosed with stage I-II cancers in both arms, since recurrence is not included in registries, with associated logistical challenges. This modified endpoint of recurrence-updated stage would not be subject to the influence of either overdiagnosis or lead-time. Furthermore, it would yield a more robust result within a shorter timeframe than if cancer-specific mortality were used as the primary outcome. In the hypothetical scenario given above, if recurrence-updated stage had been the primary outcome, then no reduction in late-stage disease would have been detected in the intervention arm, as the stage of the MCED-detected cancers would have been later updated to stage III/IV (due to recurrence) and thus the trial would correctly report a negative result.

The hypothetical situation discussed above in which every early-stage cancer detected by the MCED test is associated with micro-metastatic disease is an unlikely worst-case scenario. It is our hope that MCEDs can detect harmful cancers within a curable window of opportunity, and thus deliver a ‘paradigm shift’ in cancer outcomes. However, the possibility of micro-metastatic disease in MCED-detected cancers is a real concern, and thus trials attempting to clarify the efficacy of MCEDs must be designed to account for this phenomenon. If MCEDs do reduce the incidence of late-stage cancers, even when stage is updated for recurrence, then we can have higher confidence in their potential to actually reduce cancer deaths. This would eliminate one of the strongest threats to the success of any eventual widespread roll-out of MCED tests.

## References

[1] Neal RD, Johnson P, Clarke CA, Hamilton SA, Zhang N, Kumar H (2022). Cell-Free DNA–Based Multi-Cancer Early Detection Test in an Asymptomatic Screening Population (NHS-Galleri): Design of a Pragmatic, Prospective Randomised Controlled Trial. Cancers..

[2] Lee LYW. A moment to celebrate in our potentially revolutionary cancer blood tests trial. Accessed August 10, https://www.england.nhs.uk/blog/a-moment-to-celebrate-in-our-potentially-revolutionary-cancer-blood-tests-trial/. (2022).

[3] Chen X, Dong Z, Hubbell E, Kurtzman KN, Oxnard GR, Venn O (2021). Prognostic significance of blood-based multi-cancer detection in plasma cell-free DNA. Clin Cancer Res..

[4] Menon U, Gentry-Maharaj A, Burnell M, Singh N, Ryan A, Karpinskyj C (2021). Ovarian cancer population screening and mortality after long-term follow-up in the UK Collaborative Trial of Ovarian Cancer Screening (UKCTOCS): a randomised controlled trial. Lancet..

[5] Soong TR, Howitt BE, Horowitz N, Nucci MR, Crum CP (2019). The fallopian tube, “precursor escape” and narrowing the knowledge gap to the origins of high-grade serous carcinoma. Gynecologic Oncol..

